# Survey methodology of the geographic research on wellbeing (GROW) study

**DOI:** 10.1186/s13104-015-1379-2

**Published:** 2015-09-02

**Authors:** Catherine Cubbin

**Affiliations:** School of Social Work, The University of Texas at Austin, 1925 San Jacinto Boulevard, Mail Code D3500, Austin, TX 78712 USA

## Abstract

**Background:**

Causal inferences from survey research on health would benefit from population-based prospective survey designs. Because of decreasing survey response rates and residential mobility, however, loss to follow-up is of concern. The purpose of this paper is to describe the methodology of the geographic research on wellbeing (GROW) study and the resulting sample of women, their children, and their neighborhoods.

**Results:**

GROW (2012–2013) was designed as a follow-up mail/telephone survey of postpartum women who completed the statewide-representative Maternal and Infant Health Assessment (MIHA) baseline survey (2003–2007) in California. GROW was completed in English or Spanish by mothers whose index child from MIHA were aged 4–10 years. Its research focus is on the role of neighborhood environments on behavioral risk factors for cancer. The survey was developed based on expert guidance and extensive pilot testing and includes in-depth information on women’s and children’s health and behaviors, socioeconomic and demographic factors, psychosocial characteristics, and neighborhood perceptions, linked to objective neighborhood characteristics. The sample size for GROW is 3016 women. Response rates were 33 % of the eligible sample and 75 % of the active sample (those able to be located). GROW appears to be highly representative of its target population and its respondents lived in similar types of neighborhoods compared with all California neighborhoods.

**Discussion:**

Surveyed 5–10 years after baseline, the GROW mixed-mode methodology produced a prospective, representative sample of women with young children in California, comparing both individual and residential characteristics. The methods have implications for the 40 states and New York City that participate in CDC’s Pregnancy Risk Assessment Monitoring System, as well as other cross-sectional studies with participants’ contact information. Several recommendations for conducting similar follow-up studies with minimal loss to follow-up are provided.

## Background

Research on neighborhood effects on health, and research based on cross-sectional surveys in general, would benefit from more prospective designs [1–6]. Prospective designs could allow for assessments of selection effects, repeated measures, and temporal sequencing, making causal inferences more feasible. In addition, population-based study designs of diverse populations increases the external validity of their findings. However, decreasing response rates to mail and telephone surveys make it more difficult to minimize non-response bias and draw generalizable inferences [7–9].

The geographic research on wellbeing (GROW) study was a prospective, population-based study of a diverse sample of mothers in California designed to examine neighborhood effects on behavioral risk factors for cancer. Broadly speaking, the aim of GROW is to examine the effects of neighborhood SES and the built environment on risk factors for cancer among women and their children. GROW was a follow-up survey of participants in the Maternal and Infant Health Assessment (MIHA), California’s version of the CDC’s Pregnancy Risk Assessment Monitoring System (PRAMS), an annual, statewide-representative survey of postpartum women currently being administered in most states. In GROW, women were asked to answer questions regarding demographic, socioeconomic, psychosocial, and health-related characteristics, pertaining to themselves and their index child, as well as a rich set of neighborhood characteristics. The purpose of this paper is to describe the design and methodology of the GROW study, and to describe the GROW sample and their neighborhoods. The experience of implementing GROW may be relevant for PRAMS states, and/or other cross-sectional studies participants’ contact information at baseline, creating an opportunity to implement follow-up studies.

## Results

### Baseline survey

GROW was designed as a follow-up survey of participants in California’s MIHA survey (2003–2007), a collaborative project of the California Department of Public Health and researchers at the University of California, San Francisco. MIHA, which is very similar to PRAMS, is an annual, cross-sectional, statewide-representative survey of mothers delivering live infants in California from February to May, linked with birth certificate data. Women are eligible for MIHA if they are English- or Spanish-speaking California residents, aged 15 years or older, with singleton, twin, or triplet births, and whose addresses are recorded in birth certificates; the sample is selected according to region, education, and race/ethnicity, oversampling African Americans. Each year, MIHA surveys approximately 3500 women representing approximately 500,000 births during the same year. Self-administered surveys in English and Spanish are mailed to women starting about 8 weeks after they give birth. Telephone contact is attempted with nonresponders and those whose surveys are returned because of incorrect addresses, with a protocol that specifies multiple calls at different times of the day and days of the week. For 2003–2007, approximately 99 % of the surveys were completed between 2 and 7 months after the date of birth. Questionnaires were completed by mail for 69 % and by telephone for 31 % of respondents; 71 % of the surveys were completed in English and 29 % in Spanish. Response rates exceeded 70 % each year. The maternal characteristics of the MIHA sample are representative of all eligible births statewide [10].

### Feasibility study

In 2007, a feasibility study was completed to determine whether respondents from the baseline MIHA could be successfully located for potential follow-up studies. For three MIHA survey years (2002, 2004, 2006), a random sample of women was selected, stratified by education and language. Respondents’ contact information was used to reach them by telephone to complete a brief survey. Out of the 238 women sampled for the feasibility study, 89 % had agreed to be re-contacted in the original MIHA survey and, of those, 67 % were able to be successfully located. Results were similar by year and language; lower completion rates were observed for women with lower education. The results of the feasibility study suggested that women from MIHA could be effectively located and invited to participate in a follow-up study several years later.

### Follow-up survey

Because of budget limitations, it was not feasible to follow-up all women for GROW who were interviewed at baseline (5 years of MIHA, 2003–2007). Therefore, a decision was made to follow-up MIHA respondents from six largely urbanized counties with the highest number of respondents: Alameda, Los Angeles, Orange, Sacramento, San Diego, and Santa Clara. Respondents in these 6 counties represented 55 % of all respondents in MIHA from 2003 to 2007.

The intent for the GROW survey was to appear as similar as possible to MIHA (i.e., format, length, language, and under an 8th grade reading level). The conceptual framework guiding the survey development comes from the social ecologic and social determinants of health models, with the main aim of neighborhood-level influences on health behaviors for cancer as a core guiding principle. As well, we included some items from MIHA to assess change. The survey development process lasted 14 months from inception to printer-ready IRB-approved materials [11]. This process began with an outline of the measurement domains and a search of existing instruments. Multiple drafts of the instrument were reviewed by the project investigators to refine the survey. Six focus groups were then conducted and audio-recorded between July, 2011 and November, 2011, among mothers in California who had children in the same age range as expected in GROW. Each group contained 8–11 participants who received $75 in compensation, and varied in composition in terms of race/ethnicity, educational attainment, location, and language (“Appendix”). The structure of each group consisted of the moderator explaining the purpose of the study and focus group. Participants were then given time to complete the survey in a private location and were reconvened to discuss any items that were unclear. Focus groups were staggered over time to allow for refinement of the instrument and translation into Spanish. Finally, a convenience sample of seven women with children was used to pilot test the final mail version for timing and logic and a convenience sample of six women were interviewed by phone via computer-assisted telephone interviewing (CATI) to pilot test the phone version.

### Identifying the index child

To facilitate accurate identification of the index child (the child referred to in the baseline MIHA survey and for whom data was collected in GROW), we used two approaches: In the cover letter in the mailed survey and phone script, women were asked to refer to their daughter or son who was born shortly before they completed the MIHA survey (February–May, 2003–2007), necessitating 40 versions (sex of child by 4 months by 5 years) of the cover letters in both English and Spanish. As well, before responding to any questions about their child, women were prompted to respond about that same child referred to in the cover letter (or phone script) and, for the mailed version, whose month and year of birth was also printed on the cover of the survey itself.

The GROW study was approved by the Institutional Review Boards at the University of Texas at Austin, the University of California, San Francisco, and the California Department of Public Health; all participants gave informed consent.

### Data collection

#### Identifying eligible women

**Fig. 1:**
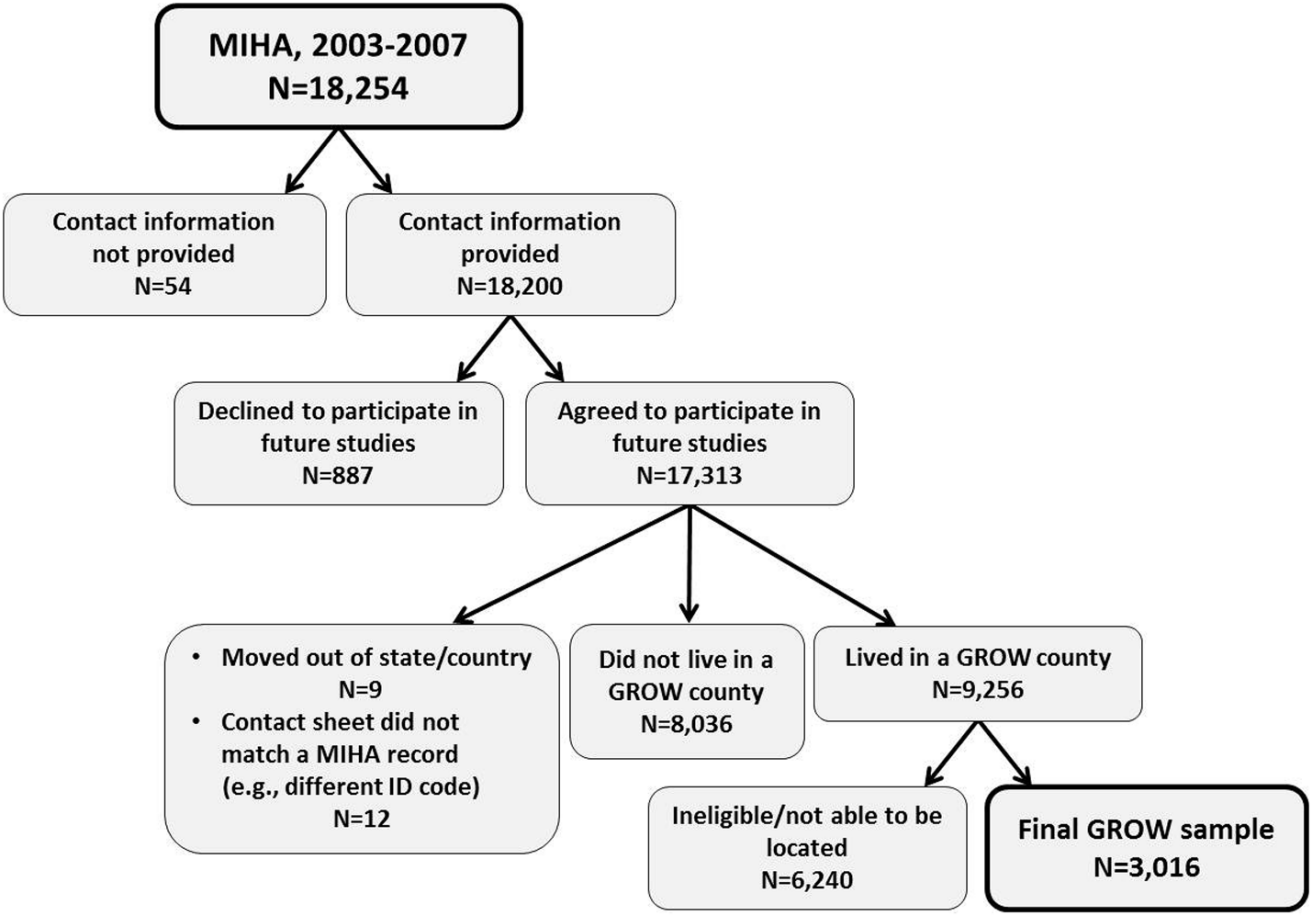
Summary flowchart of sample selection for GROW

MIHA respondents were asked to provide their address to receive their gift card, as well as whether they would be willing to participate in a future survey. If they said yes, they were then asked to provide their home and work numbers and names/addresses/phone numbers of two people who did not live with them and would know where to find them. Contact information was available for 18,200 women in MIHA 2003–2007. Of these women, we found that 51 % (n = 9256) were eligible for GROW (agreed to be re-contacted and lived in one of the six counties). Figure 1 presents a summary flowchart of the sample selection process.

#### Initial recruitment

In February, 2012, the 9256 addresses for GROW-eligible participants were run against the National Change of Address database to determine if any addresses had changed. A letter was then mailed to both notify women that they would soon be receiving a survey in the mail and to ascertain undeliverable addresses before the actual mailing. For deliverable addresses, the questionnaire packets were mailed several weeks later in the language determined by the one in which women completed the MIHA survey and a reminder postcard was mailed several weeks after that initial mailing. Tracing and re-mailing (when requested) was performed continuously. Telephone surveys (for those with undeliverable addresses and nonrespondents to the mailed surveys) were conducted continuously between May, 2012 and May, 2013, using all contact information provided by MIHA respondents and through tracing. To increase response, in November, 2012, and then in January, 2013, additional contact information was obtained for nonrespondents who were participating in the Women, Infants, and Children (WIC) program through an approved data linkage request. Second, in March, 2013, a postcard was mailed notifying women of a doubling of the gift card incentive and an additional raffle to win an iPad, iPod touch, or a children’s bicycle. Women were asked to respond to approximately 80 questions regarding demographic, socioeconomic, psychosocial, and health-related characteristics, pertaining to themselves and their index child, as well as a rich set of neighborhood characteristics. The final dataset was completed in September, 2013. Fifty-six percent completed the survey by phone, and 73 % completed it in English. Among mail respondents, 77 % were in English and, among phone respondents, 57 % were in English. On average, the survey took 44 min to complete.

#### Neighborhood variables

Neighborhood (census tracts) data were from the Neighborhood Change Database (NCDB), published by Geolytics, Inc., and from the American Community Survey 2005–2009 (ACS). All data in the NCDB were from the US Bureau of the Census (1970, 1980, 1990, and 2000 decennial censuses) and, because geographic tract boundaries change over time, the data were recalculated and weighted to correspond to census 2000 boundaries so that data represented the same geographic areas over time [12]. The ACS is an ongoing annual survey conducted by the US Census that collects data similar to that obtained in the decennial census; we used data combined across 2005–2009, which provides information at the census tract level. Census tract variables are linked to the GROW database via FIPS geocodes.

#### Built environment data

Built environment data were available from business license data, parks data, farmer’s markets data, transit data, and street networks. We obtained data for 80,153 business licenses in the 6 GROW counties from Infogroup (http://www.infogroup.com) in September, 2011. These businesses included dollar stores, pharmacies, all food-related businesses (convenience stores, grocery stores, fast food restaurants), on- and off-premise alcohol outlets, tobacco retailers, gyms and health clubs, banks and check cashing businesses, and cleaners (laundry), based on 4-digit Standard Industrial Classification (SIC) codes. License information is gathered from multiple sources and verified on an annual basis via telephone calls. Of these, we excluded 5948 records because they did not fall into one of our selected categories (e.g., catering businesses, coffee roasters) and 16 duplicate records. Of the remaining businesses, 555 listed a P.O. Box as their primary address and for those businesses, we used the secondary address as the physical location (based on calling a 5 % sample of these businesses and determining that the secondary address was actually the physical location for more than 90 % of those businesses). Through this process, we excluded 44 businesses that had either closed or were not a physical business. Our final sample was 74,145 records. Business license data was supplemented by parks data from the California Protected Areas Database (CPAD) and from county parks and recreation departments, farmer’s markets data from the California Federation of Certified Farmer’s Markets, transit data (e.g., bus stops) from county and city governments, and street network data from Census TIGER files.

#### Final data preparation

Weights were created to produce data that were representative of the birth file and original MIHA sample in the six GROW counties, and a sampling fraction file was created to make a minor finite population correction to the standard errors for analyses. Data cleaning included a comparison of child’s date of birth from the birth certificate and GROW, resulting in 13 children where the mother may have responded for a child other than the index child. For child’s weight and mother’s height and weight, improbable values based on CDC recommendations were set to missing. To decrease the number of missing data for mother’s BMI, a manual review of the mailed surveys was completed and height data was imputed from MIHA or the birth certificate, when available. For Latinas, the group with adequate sample sizes to stratify by nativity, those with missing place of birth information based on MIHA self-report was imputed from the birth certificate. Write-in or phone responses for “other” on three survey items were manually reviewed and recoded. Cleaned addresses provided by GROW respondents were sent to Mapping Analytics (http://www.mappinganalytics.com/) for geocoding; their geocoding has demonstrated accuracy [13]. Respondents were assigned to census tracts (based on 2000 census geography) and latitude/longitudes, and each assignment was given a “match code” to indicate level of accuracy. Tracts and point data were then used to merge in census data and calculate geographic variables.

#### General summary and response rates

The GROW sample size is 3016; 90.3 % of respondents still lived in one of the six GROW counties. The geocoding accuracy to census tracts was very high at 97 %. There was an average of 1.4 respondents per tract (range 1–9); 90 % of tracts contained only 1 or 2 GROW respondents. Missing values were less than 8 % for all items except income (10.3 %).

Of the 9256 women who were initially identified as eligible to be in the sample, 0.7 % were determined to be subsequently ineligible for a number of reasons (e.g., out of the US, in a correctional facility, no longer living) and 56 % were never able to be located (i.e., did not reply to mailings or phone calls so that contact could not be made), resulting in an “active” sample of 4026. Thus, the response rate among all those initially eligible was 32.6 %, but among the active sample, it was 74.9 %. We found response rates that were quite similar across MIHA characteristics: By baseline year (72.9–77.8 %, lowest for 2003, highest for 2007), language (74.0 % for English vs. 77.6 % for Spanish), county (73.3–81.8 %, lowest for Santa Clara, highest for Alameda), income (71.9–78.4 %, lowest for those with missing income, highest for those with over 400 % of the federal poverty level), education (70.7–79.4 %, lowest for those with some college education, highest for college graduates), and race/ethnicity (70.2–77.3 %, lowest for US-born Latinas, highest for Whites and immigrant Latinas).

#### Nonresponse bias and external validity

SAS software (Cary, North Carolina) was used for all descriptive analyses. Comparing “eligible” and “active” nonrespondents to GROW respondents (Table 1), there were significant differences in distributions by county, baseline MIHA year (eligible nonrespondents), and sociodemographic characteristics. The GROW sample had higher proportions of respondents from Alameda and Sacramento counties and lower proportions from Los Angeles county. Higher proportions of GROW respondents were from the 2007 MIHA, were White, and had higher incomes and educational attainments, as would be expected (Table 1).

**Table 1:** Comparison of GROW respondents (N = 3016) to active nonrespondents (N = 1010), and to all eligible nonrespondents (N = 6240), unweighted

	GROW respondents	Active^a^ non-respondents	P value	Eligible^b^ non-respondents	P value
Language, % English	72.8	76.5	0.020	66.6^a^	<0.0001
County, %					
Alameda	9.2	6.1	0.006	7.1	0.0003
Los Angeles	43.8	47.3		47.1	
Orange	14.8	15.9		15.5	
Sacramento	8.1	6.0		6.9	
San Diego	15.3	15.0		15.4	
Santa Clara	8.8	9.6		8.1	
Baseline MIHA year, %					
2003	17.6	19.5	0.208	22.3	<0.0001
2004	17.9	18.0		20.6	
2005	18.8	19.6		20.6	
2006	21.5	22.1		20.0	
2007	24.3	20.8		16.4	
Race/ethnicity, %					
African American	11.5	13.5	0.002	17.7	<0.0001
Asian/Pacific Islander	9.7	11.0		10.1	
Latina, immigrant	28.5	25.1		34.6	
Latina, US-born	15.2	19.4		16.7	
White	32.8	28.8		18.9	
Family income, % of federal poverty level					
0–100 %	23.2	28.2	0.019	38.6	<0.0001
101–200 %	16.6	20.0		19.9	
201–300 %	9.9	8.7		7.6	
301–400 %	8.4	7.1		5.3	
401+ %	31.7	26.6		17.3	
Missing	10.3	9.3		11.3	
Educational attainment, %					
Did not complete high school	16.7	17.0	<0.0001	22.7	<0.0001
High school graduate/GED	21.4	24.3		30.0	
Some college	23.4	28.9		25.5	
College graduate or more	38.1	29.4		21.2	

p values are based on a Chi square statistic

^a^Active = able to be located

^b^Eligible = women who gave permission to be re-contacted and lived in the six counties

However, comparing the weighted GROW sample to the weighted MIHA sample and the Target population of all women in California who gave birth during the relevant time period (Table 2), GROW appears to be highly representative in terms of important sociodemographic characteristics (i.e., race/ethnicity, birthplace, age, education).

**Table 2:** Comparison of characteristics of GROW respondents (2012–2013), MIHA (2003–2007) respondents, and the Target Population (2003–2007), six counties

Birth certificate variable	Characteristics of women in GROW, MIHA, and in the target population				
	All grow counties, %				
	GROW_Unweighted_	GROW_Weighted_	MIHA_Unweighted_	MIHA_Weighted_	Target^a^
Total, N	2996^b^	1,531,072	9954	1,531,072	1,531,072
Race/ethnicity (%)					
Missing	1.8	2.0	1.8	1.7	1.7
African American	11.6	6.5	15.3	6.4	6.3
Asian/Pacific Islander	9.7	14.5	11.0	14.9	15.1
Latina	43.7	51.7	47.6	51.5	51.8
White, non-Latina	32.9	24.8	24.0	25.2	24.8
Other	0.4	0.6	0.4	0.5	0.3
Birthplace (%)					
Missing	0.0	0.0	0.1	0.1	0.1
Foreign-born	40.8	51.4	46.1	51.7	52.1
US-born	59.2	48.5	53.8	48.2	47.8
Age, years (%)					
15–19	5.7	7.4	8.9	8.1	8.4
20–24	16.7	20.8	21.6	21.1	21.0
25–29	24.3	27.2	25.8	26.3	26.0
30–34	28.0	24.0	25.2	25.8	26.0
≥35	25.3	20.7	18.6	18.7	18.6
Education (%)^c^					
Missing	2.5	2.3	1.8	1.7	1.6
≤8th grade	9.0	11.1	9.3	9.8	9.9
Some high school	14.9	17.2	20.0	18.9	18.7
High School graduate	17.8	22.5	21.9	23.2	23.6
Some college or more	55.8	47.0	47.0	46.4	46.2

^a^All births in the six GROW counties

^b^GROW respondents exclude 20 women who were sampled into GROW who did not reside in a GROW county at birth, but did reside in a GROW county at MIHA

^c^From 2003 to 2005, education was coded in number of years; this was recoded to be consistent throughout the study period

#### Characteristics of the GROW sample

Selected characteristics are presented in Table 3. On average, mothers were aged 36 and index children were aged 7. Just under half of the children were girls. Mothers and children were racially/ethnically diverse. The large majority of mothers were married or living with a partner. An average of nearly three children resided in the household and nearly 20 % had moved at least twice in the previous 5 years. Respondents were diverse socioeconomically based on family income and mothers’ educational attainment. Just over half of the mothers were employed the previous 2 weeks (most working full-time), and just over half were renting their homes. Nearly a quarter of mothers reported food insecurity during the past year (operationalized with a 6-item food insecurity scale developed by researchers at the National Center for Health Statistics and that include questions referencing the last 12 months and querying mothers on issues such as: “The food I bought just did not last, and I did not have money to get more”, “I could not afford to eat balanced meals”, and “Cut size of meals or skipped meals because there wasn’t enough money for food” [14]; we coded households who answer affirmatively to at least 2 of the items as food insecure).

**Table 3:** Selected characteristics of the GROW sample, weighted, N = 3016

Demographic characteristics	
Mother’s age, years (mean, range)	36.1 (20–57)
Child’s age, years (mean, range)	6.9 (4–10)
Child’s sex, % girls	48.6
Mother’s race/ethnicity (%)	
African American/Black	6.6
American Indian/Alaskan Native or other	0.6
Asian/Pacific Islander	14.8
Latina, immigrant	36.9
Latina, US-born	15.9
White	25.3
Child’s race/ethnicity (%)	
African American/Black	5.1
American Indian/Alaskan Native	0.2
Asian/Pacific Islander	10.6
Latina/o	56.3
Multiple race/ethnicity	7.1
White	20.8
Mother’s marital status (%)	
Married	70.1
Living with someone as if married	13.3
Separated/divorced/widowed	6.9
Single/never married	9.8
Number of children in household, all ages (mean, range)	2.8 (0–14)
Residential mobility, times moved in past 5 years (%)	
0 times	62.6
1 time	19.4
2–5 times	16.8
>5 times	1.3
Socioeconomic characteristics	
Family Income (% of federal poverty level) (%)	
0–100 %	27.3
101–200 %	18.0
201–300 %	10.2
301–400 %	7.4
401+ %	26.0
Missing	11.2
Mother’s educational attainment (%)	
8th grade or less	10.8
Some high school	9.8
High school graduate or GED	22.3
Some college	22.8
College graduate or more	34.3
Mother’s employment status in past 2 weeks (%)	
Unemployed	46.3
Worked part-time (<40 h/week)	23.4
Worked full-time (40+ h/week)	30.3
Housing tenure (%)	
Home owner (with or without a mortgage)	44.7
Renter	53.0
Other (lived in but pay no rent)	2.3
Mothers with food insecurity (%)	23.4
Neighborhood characteristics	
Length of time in neighborhood (%)	
<1 year	8.3
1–5 years	26.8
6–10 years	31.9
>10 years	33.0
Social cohesion in neighborhood (mean, range)	14.7 (5–20)
Neighborhood safety from crime (% very/somewhat unsafe)	15.4
Mother’s Psychosocial characteristics	
Depressive symptoms in past 12 months (% yes)^a^	19.9
Emotional support (% yes)^b^	93.3
Practical support (% yes)^c^	92.4
Financial support (% yes)^d^	79.1
Number of friends (mean, range)	7.9 (0–200)
Health-related characteristics	
Child’s	
Activity limitation in past 30 days (% >1 day)	5.5
Days of physical activity for at least an hour (% every day)	28.6
Percentile of weight for age (mean, range)	54.3 (0–99.99)
Daily consumption of fruit (%)	65.7
Daily consumption of vegetables (%)	49.7
Mother’s	
Activity limitation in past 30 days (% >1 day)	16.4
Health status as fair/poor (%)	21.8
Sedentary physical activity (%)	40.2
BMI (mean, range)	26.8 (14.9–62.2)
Daily consumption of fruit (%)	48.5
Daily consumption of vegetables (%)	57.9

^a^2 weeks or longer when respondent felt sad, empty, or depressed for most of the day

^b^Someone to turn to if respondent needed someone to comfort or listen to her

^c^Someone to help with practical help, like getting a ride, help with shopping/cooking a meal, or watching her children for a short time

^d^Someone to turn to if respondent needed extra help financially, like help paying for some bills, the rent or mortgage, or food that she needed

Before responding to questions about their neighborhoods, women were given the definition of a “neighborhood” used in the GROW survey, i.e., the “general area around your home where you might spend time and visit with neighbors or take a short walk. It may also include places where you shop and other local businesses, churches, or schools.” About two-thirds of mothers had lived in their neighborhood at least 6 years and their perception of neighborhood social cohesion was moderately high (operationalized with a summary scale using 5 questions asking how respondents feel about their neighborhood, i.e., how connected neighbors feel to one another, how willing people are to help their neighbors, how well people get along, whether people share the same values, and to what extent people can be trusted; responses range from strongly agree = 4 to strongly disagree = 1). However, about 15 % of women felt their neighborhood was very or somewhat unsafe due to crime.

Although one-fifth of mothers reported having depressive symptoms in the past year, they also reported high social support (79–93 %). While only 5.5 % of children had their activities limited for at least a day during the past month, daily physical activity and fruit and vegetable consumption was relatively low, and average weight for age was over the 50 percentile at 54.3 %, indicating that they were slightly overweight compared with national growth chart distributions. About three times as many mothers (16.4 %) compared with children were limited in their activity and over one-fifth reported their health status as fair or poor. Two-fifths of mothers were sedentary and daily consumption of fruit and vegetables was 49–58 %. Mean BMI was in the overweight category at 26.8 kg/m^2^.

#### Characteristics of the GROW sample’s neighborhoods

Table 4 presents census tract characteristics, for all tracts in California compared with tracts where GROW respondents lived. The characteristics were remarkably similar aside from the pattern that GROW tracts appeared to have higher population density and racial/ethnic heterogeneity, as expected, given that the sample was selected from large, urbanized counties.

**Table 4:** Selected census tract characteristics, California tracts (N = 7049) and GROW tracts, (N = 1906), American Community Survey, 2005–2009

	California mean (SD)	GROW mean (SD)
Population density, people per square kilometer	3130 (3435)	3739 (3262)
Median family income, $	74,142 (36,718)	75,469 (36,902)
Median housing value, $	504,288 (222,210)	527,036 (197,062)
Percent poor persons	13.1 (10.5)	13.0 (10.3)
Percent unemployed	8.1 (4.8)	7.8 (3.9)
Percent who did not graduate from high school	20.6 (16.9)	21.9 (18.0)
Percent crowded housing, >1 person per room	8.9 (10.3)	10.7 (11.5)
Percent under age 18	25.1 (8.0)	26.5 (6.9)
Percent of children in single parent household	27.1 (15.8)	27.2 (15.2)
Racial/ethnic concentration		
Percent African American/Black	6.0 (10.3)	7.5 (12.3)
Percent Asian	12.3 (14.5)	13.9 (14.3)
Percent Hispanic/Latino	34.9 (26.6)	38.2 (27.7)
Percent White	43.9 (27.8)	37.7 (27.5)
Percent foreign-born	26.5 (15.2)	29.8 (14.5)

Excludes census tracts for women who did not live in California at the time of the GROW survey

Selected built environment characteristics are presented in Table 5, and are based on residential addresses of women in the GROW sample who still lived in the six counties. On average, the closest park to a respondent’s address was about a half kilometer away and the total area of all parks within a half mile of her address was about a tenth of a kilometer squared. Also, GROW respondents had more fast food restaurants (3.6, standard error 0.08) than grocery stores (2.1, standard error 0.06) or tobacco outlets (1.7, standard error 0.04) near their address, and the closest fast food restaurant (just under a kilometer) was closer than the closest grocery store (1.1 km) or tobacco outlet (1.2 km).

**Table 5:** Selected Built Environment characteristics, GROW respondents living in six California counties, N = 2637

	Mean (SE)
Parks	
Straight line (Euclidian) distance to closest park (kilometers)^a^	0.458 (0.007)
Area of parks within a 0.5 mile buffer (square kilometers)^a^	0.102 (0.003)
Tobacco outlets	
Street network distance to closest tobacco outlet (kilometers)	1.209 (0.030)
Mean number of all tobacco outlets within a 0.5 mile buffer	1.7 (0.04)
Mean straight line (Euclidian) distance of all tobacco outlets within a 0.5 mile buffer (kilometers)^b^	0.530 (0.004)
Grocery stores	
Street network distance to closest grocery store (kilometers)	1.129 (0.023)
Mean number of all grocery stores within a 0.5 mile buffer	2.1 (0.06)
Mean straight line (Euclidian) distance of all grocery stores within a 0.5 mile buffer (kilometers)^c^	0.538 (0.004)
Fast food restaurants	
Street network distance to closest fast food restaurant (kilometers)	0.988 (0.023)
Mean number of all fast food restaurants within a 0.5 mile buffer	3.6 (0.08)
Mean straight line (Euclidian) distance of fast food restaurants within a 0.5 mile buffer (kilometers)^d^	0.562 (0.003)

Excludes 103 women with inaccurate tract geocodes

^a^Excludes parks less than 0.1 acre

^b^Tobacco outlets include tobacco shops and convenience stores; mean distance is for the 1647 respondents with at least 1 outlet within 0.5 mile of her residence

^c^Grocery stores include full-service markets, fruit and vegetable markets, and farmer’s markets; mean distance is for the 1688 respondents with at least 1 store within 0.5 mile of her residence

^d^mean distance is for the 1920 respondents with at least 1 restaurant within 0.5 mile of her residence

## Discussion

The GROW survey design and methodology produced a representative sample of women with young children in California. Although the response rate of the eligible participants was lower than was anticipated from the feasibility study, this may have been expected given the lag in time between the feasibility study and survey administration, i.e., more time elapsed, making women possibly harder to locate. With the appropriate weights, however, a representative sample was obtained with little missing data. Furthermore, characteristics of the sample are similar to what would be expected compared with other population-based data sources. The success of the GROW study was primarily due to extensive contact information that was available and to the cooperation from the California Department of Public Health. Manuscripts are currently in process, focusing on a number of methodological and health inequalities-related topics, including residential mobility, neighborhood change and selection effects, depressive symptoms, smoking cessation, dietary habits, physical activity, and food insecurity.

Strengths of the study include the high quality baseline dataset from which to draw the eligible sample for follow-up, a strong collaborative team to administer the survey, and the resulting rich source of data on health, socioeconomic, and neighborhood characteristics for a diverse sample of women and their children. Limitations include a lower than anticipated response rate, primarily because of the lack of current contact information. Nonrespondents were more likely to be women of color or of lower socioeconomic status compared with respondents. The study is also limited in terms of having self-reported information only, proxy reports for children, and underrepresentation of rural areas.

In conclusion, prospective, representative mail/telephone surveys of a diverse sample of women can be successfully collected based on existing contact information from a baseline survey collected 5–10 years prior. This has potential implications for the 40 states and New York City that participate in PRAMS, as well as other cross-sectional studies with participants’ contact information. Currently, four PRAMS states have on-going follow-up of their samples, but not as far out as 5–10 years.

Recommendations for conducting similar follow-up studies include the following. First, ethical collection and secure storage of accurate and extensive contact information on baseline surveys is essential. Researchers often routinely collect respondent contact information for disbursement of incentives; contact information for additional people who would know how to reach the eligible sample is recommended to minimize loss to follow-up. As well, it is recommended to ask respondents if they would allow permission to be re-contacted for a potential follow-up study (only 4.9 % of the MIHA sample did not give permission). Asking for permission could help to establish trust in research as well as possibly increase response. In the GROW survey, permission and additional contact information was asked in anticipation of a potential third survey to establish a longitudinal sample when GROW children are older. Third, the GROW study benefited from a team with extensive experience in survey administration (survey design and implementation; highly skilled, well-trained, bilingual interviewers; tracking procedures, etc.). Careful attention must be paid to language, ordering, format, skip patterns, translation, and training/monitoring. The pre-testing phase was especially critical in finalizing the survey. The relative lack of missing data, positive comments from survey respondents, as well as feedback from the telephone interviewers were indicators that the survey worked well. Finally, strong partnerships with collaborators is also necessary: With GROW, this proved essential for the linkage of eligible respondents with administrative data (i.e., WIC records) to locate more women and increase response. Response rates would likely have been higher, however, if social security numbers had been permitted for additional linkages to existing databases. It is hoped that the experience of GROW will be useful to other researchers wishing to conduct similar prospective studies.

## Appendix

See Table 6.

**Table 6:** Focus group composition and location, geographic research on wellbeing (GROW) study

Date	Number of participants	Location	Language	Race/ethnicity	Socioeconomic status
07/05/11	11	Oakland	English	Mixed	Mixed
07/25/11	11	Oakland	English	Mixed	Mixed
08/12/11	8	San Diego	English	African American	Low
09/09/11	9	Los Angeles	Spanish	Latina	Low/moderate
10/19/11	10	Oakland	Spanish	Latina	Low
11/04/11	10	Oakland	English	Latina	Mixed

## Abbreviations

MIHA: Maternal and Infant Health Assessment
GROW: geographic research on wellbeing
PRAMS: Pregnancy Risk Assessment and Monitoring System
CATI: computer-assisted telephone interview
NCDB: Neighborhood Change Database
ACS: American Community Survey
